# Testing effects of vapor pressure deficit on fruit growth: a comparative approach using peach, mango, olive, orange, and loquat

**DOI:** 10.3389/fpls.2023.1294195

**Published:** 2023-12-20

**Authors:** Alessandro Carella, Roberto Massenti, Riccardo Lo Bianco

**Affiliations:** Department of Agricultural, Food and Forest Sciences, University of Palermo, Palermo, Italy

**Keywords:** cell division, cell expansion, fruit diameter, fruit gauge, fruit maturation, fruit water relations, precision horticulture, proximal sensing

## Abstract

Determining the influence of vapor pressure deficit (VPD) on fruit growth is a key issue under a changing climate scenario. Using a comparative approach across different fruit tree species may provide solid indications of common or contrasting plant responses to environmental factors. Knowing fruit growth responses to VPD may also be useful to optimize horticultural management practices under specific atmospheric conditions. Climate data to calculate VPD and fruit relative growth rates (RGR) by fruit gauges were monitored in peach at cell division, pit hardening and cell expansion stages; in two mango cultivars at cell division, cell expansion and maturation stages; in two olive cultivars, either full irrigated or rainfed, at early and late cell expansion stages; in ‘Valencia’ orange at early and late cell division stage, before and after mature fruit harvest; in loquat at cell expansion and maturation stages. At the fruit cell division stage, sensitivity of fruit growth to VPD seems to vary with species, time, and probably soil and atmospheric water deficit. ‘Keitt’ mango and ‘Valencia’ orange fruit growth responded to VPD in opposite ways, and this could be due to very different time of the year and VPD levels in the monitoring periods of the two species. At pit hardening stage of peach fruit growth, a relatively weak relationship was observed between VPD and RGR, and this is not surprising as fruit growth in size at this stage slows down significantly. A consistent and marked negative relationship between VPD and RGR was observed at cell expansion stage, when fruit growth is directly depending on water intake driving cell turgor. Another behavior common to all observed species was the gradual loss of relationship between VPD and RGR at the onset of fruit maturation, when fruit growth in size is generally programmed to stop. Finally, regardless of fruit type, VPD may have a significant effect on fruit growth and could be a useful parameter to be monitored for tree water management mainly when the cell expansion process prevails during fruit growth.

## 1 Introduction

In Mediterranean environments, high radiation and air temperature associated with high vapor pressure deficit (VPD) during summer affect both plant water status and production in terms of yield and quality (Somboonkaew and Terry, 2010; Bardi et al., 2022; Noh and Lee, 2022). According to some researchers the global increase in VPD leads to a decrease in plant productivity (Yuan et al., 2019). Increased VPD often causes a closure of leaf stomata resulting in decreased rates of photosynthesis (Fletcher et al., 2007). The transpiration rate of fruits, particularly for those that are well-exposed, is mainly determined by the VPD between the air and the evaporating surface, causing continuous diameter fluctuations. These variations, mainly that of daily contraction, are usually interpreted as elastic changes in tissue volume (Léchaudel et al., 2007). Indeed, the daily diameter variations of fleshy fruits involve a balance between water intake, on one side, and withdrawal through vascular tissue and losses by transpiration, on the other side.

In the early stage of fruit development, at green stage, when the chlorophyll content is high, fruit stomata are sensitive as in leaves. High VPD gradually induce stomatal closure, leading to a reduction in the rate of CO_2_ assimilation in the fruit (Blanke and Lenz, 1989; Garrido et al., 2023). These mechanisms should vary in the case of fruits without stomata, such as tomato (Rančić et al., 2010), where water losses from the fruit occur mainly by cuticular transpiration. Transpiration flow in this case may depend on the composition of the cutin and waxy layer and on the concentration of microscopic polar pores (Zarrouk et al., 2018; Fich et al., 2020; Garrido et al., 2023). In peach, it was observed that transpiration, in response to daytime environmental conditions, decreases fruit water content along with a reduction in turgor and water potential, causing fruit shrinkage. Carbohydrates reaching the fruit from the phloem may also accumulate in the vacuole reducing osmotic and ultimately water potential. Later in the day, this, along with resumed xylem flow, attracts greater amounts of water into the fruit from the xylem than are lost through transpiration causing fruit expansion (Morandi et al., 2007b). Drought and VPD, due to the rapid stomatal closure also have a negative effect on photosynthesis (Léchaudel et al., 2013), and thus on the accumulation of dry matter in the fruit. In olive fruits, 10 to 30% of fresh weight (FW) may be that of the endocarp, depending on the cultivar (Del Rio and Caballero, 2008), crop load (Lavee and Wodner, 2004) and water availability (D’Andria et al., 2004; Lavee et al., 2007). In olive under well-watered conditions, Fernandes et al. (2018) showed that fruit contraction was mainly driven by high VPD. In ‘Keitt’ mango, VPD was the main driving force determining fruit diameter fluctuations (Carella et al., 2021). Also peach fruit shrinkage and growth have been related to high VPD and fruit transpiration (Morandi et al., 2007b; Morandi et al., 2010). An inverse relationship between VPD and fruit relative growth rate was found in ‘Valencia’ orange when data over a 5-year period were pooled together (Mossad et al., 2018).

Fruit development also plays a key role. In mango, the skin, the flesh and the stone have specific compositions that appear to accumulate water and dry matter at different rates, depending on environmental conditions (Léchaudel et al., 2007). Most fruits can have a sigmoid or double sigmoid growth pattern. These patterns are divided into development stages: cell division, pit hardening (in the case of fruits with double sigmoid pattern), cell expansion, and ripening. Cell division is a high energy demanding process (Li et al., 2012), due to the fast cell division rate in fruit tissues. Thus, ensuring an adequate supply of carbohydrates becomes crucial during this stage. Carbohydrates translocated into the fruit are mainly imported from actively photosynthesizing leaves through the phloem (Génard et al., 2008; Carella et al., 2021). Following the initial stage, fruits enter a phase of linear growth, which primarily involves the expansion of pulp cells caused by water uptake driven by osmotic gradients. This stage is significantly influenced by daily fluctuations in temperature, relative humidity, and vapor pressure deficit (VPD), as these factors play a crucial role in regulating fruit transpiration (Lescourret et al., 2001). Specifically, daily VPD fluctuations drive fruit enlargement during the night and shrinkage during the day. During pit-hardening stage, fruit growth rate is minimal or null (Rahmati et al., 2015), therefore the fruit should respond minimally to VPD changes. The final stage of fruit development is the ripening. In this stage, the fruit reaches sufficient physiological and sexual maturity to be detached from the parent plant, as described in Grierson (2002). At this stage, significant changes in the texture, flavor, and color of the fruit occur, both internally and externally (Lakshminarayana, 1973; Giovannoni, 2001), and the fruit tends to isolate itself from environmental parameters (Morandi et al., 2006; Nordey et al., 2015; Carella et al., 2021).

Several works have shown that the fruit ripening process, in terms of sugar accumulation in the pulp and skin coloration, is markedly influenced and regulated by biotic factors, such as genetic differences and crop load, and by abiotic factors, such as orchard management, ambient temperature and relative humidity, and water availability (Corelli-Grappadelli and Lakso, 2004; Gucci et al., 2009; Hammami et al., 2011). Carbon limitations caused by environmental stress during the early stages of grape berry growth may restrict berry size but do not affect the progression of ripening. On the contrary, if such limitations occur after the lag phase of berry growth, they appear to have an impact on fruit ripening (Keller, 2010).

In climacteric fruits like peaches and mangoes, the peaks in respiration rate and ethylene biosynthesis are reached during the ripening stage. In contrast, in non-climacteric fruits like loquats, oranges and olives, respiration rate and ethylene biosynthesis tend to decrease gradually (Gamage and Rahman, 1999; Rooban et al., 2016). Fruit respiration rate is also dependent on changes in temperature and relative humidity. As temperature increases and relative humidity decreases, respiration rate increases accelerating ripening phenomena (Paul et al., 2012). For these reasons, during fruit ripening, climacteric and non-climacteric fruits may exhibit different responses to environmental conditions.

VPD may be an indirect estimator of water loss in plants, and along with other parameters may provide more accurate information on irrigation scheduling. The non-destructive and continuous monitoring of changes in fruit growth is one of the parameters on which precision agriculture is based, and this is possible using fruit gauges (Morandi et al., 2007a). Fruit diameter variation by fruit gauges may represent an indirect indicator of plant water status (Lo Bianco and Scalisi, 2019; Scalisi et al., 2019a; Carella et al., 2021), and this type of indicators is needed to avoid permanent stress effects (Tomkiewicz and Piskier, 2012; Fernández, 2014; Marino et al., 2021; Massenti et al., 2022). Monitoring of fruit growth by following the diurnal fluctuation of diameter has been studied in several species, like peach and nectarine (*Prunus persica*) (Morandi et al., 2008; Scalisi et al., 2019b), plum (*Prunus domestica*) (Corelli Grappadelli et al., 2019), apple (*Malus domestica*) (Boini et al., 2019), orange (*Citrus sinensis*) (Grilo et al., 2019), olive (*Olea europaea*) (Marino et al., 2021), mango (*Mangifera indica* L.) (Carella et al., 2016) and also cladode growth in *Opuntia ficus-indica* (Scalisi et al., 2016). The aim of this work was to determine the influence of VPD on fruit growth rates, measured continuously with fruit gauges, using a comparative approach across different fruit tree species. Studying responses across different species like peach, mango, olive, orange, and loquat may serve as a powerful indicator of common or contrasting mechanisms regulating fruit growth. Ultimately, knowing species-specific VPD levels or thresholds that cause changes in fruit growth may be useful to optimize horticultural management practices under specific atmospheric conditions.

## 2 Materials and methods

### 2.1 Peach

The trial was conducted on late-ripening ‘Tardivo 2000’ peach (*Prunus persica* L.) grafted onto ‘GF 677’ rootstock in a commercial orchard of the Ecofarm company located near Riesi, in south Sicily, Italy (37.25724 N, 14.11922 E), at 330 m a.s.l., from June to September 2022. Ten 12-year-old peach trees trained to small vase and spaced at 6 x 4 m were selected for the experiment. The trial area was located on a sloping, medium-textured sandy loam soil (pH 7.3) with low active carbonates. All plants received the same conventional cultural management, including drip irrigation and fertilization. Trees were irrigated with a seasonal volume of 1443 m^3^ ha^-1^. Fruits were thinned to 1 every 15 cm of shoot on 4 June, before pit hardening.

### 2.2 Mango

The experiment was carried out in a commercial orchard of the Cupitur farm located near Caronia (38°03’ N, and 14°30’ E) at 5 m a.s.l. in northeastern Sicily (Italy) from July to October 2019. Mango (*Mangifera indica* L.) trees were protected by windbreaks made of cypress plants (*Cupressus sempervirens* L.), and nonwoven fabric windbreaks supported by 5-m-tall wooden posts. The trial was conducted on six 15-year-old mango trees, three of cv Keitt (late-season ripening) and three of cv Tommy Atkins (early- to mid-season ripening), grafted onto Gomera-3 mango rootstock, with crop loads of 1.3 and 0.7 fruits cm^-2^ of TCSA, respectively. Trees were trained to globe-shaped canopies, reaching 2.5–3 m in height, and spaced at 5 x 4 m. The soil was a loose sandy loam. Trees were fertilized and irrigated through a drip system with a seasonal volume of 3300 m^3^ ha^-1^. Two light pruning operations were carried out, one at the end of winter, before the start of vegetative growth, and one after fruit harvest.

### 2.3 Olive

The trial was carried out in summer 2016 in a high-density (6 × 3 spacing) olive (*Olea europaea* L.) orchard located near Sciacca, in South-western Sicily (37°29’ N and 13°12’ E, 138 m a.s.l.). Three-year-old own-rooted trees were trained to “free palmette” along North-South-oriented hedgerows. Sicilian cultivars Nocellara del Belice (NB) and Olivo di Mandanici (MN) were selected for their different fruit characteristics and vigor. The soil was a sandy clay loam (60% sand, 18% silt, and 22% clay) with pH of 7.7 and<5% of active carbonates. Trees were regularly fertilized and pruned according to conventional practices. Two irrigation levels were imposed to generate a large variability in tree water status: full irrigation (FI, 100% ETc) and rainfed (0% of FI). FI trees were irrigated through a drip system with a total volume of 640 m^3^ ha^-1^, while rainfed trees received 189 mm of rainwater.

### 2.4 Orange

The study was conducted on adult orange trees (*Citrus sinensis* L. Osbeck, cv Valencia) grafted onto sour orange (*Citrus aurantium* L.) in an experimental orchard located at the Department of Agricultural, Food and Forest Sciences, University of Palermo, Italy (30’06 N, 13’21 E), at 31 m a.s.l., in spring 2014. Trees were trained to globe-shaped canopies, reaching 2.5–3 m, and spaced at 4 × 4 m. Micro sprinkler irrigation was applied in 26 events at 2- to 4-day intervals, during the period between June and September. The total irrigation volume was 3870 m^3^ ha^-1^.

### 2.5 Loquat

In April 2023, 12 adult trees of the Sicilian loquat (*Eriobotrya japonica* Lindl.) cultivar Nespolone di Trabia were selected in a terraced loquat orchard located in Ciaculli, near Palermo (Italy, 38°06’ N, 13°41’ E) at 204 m a.s.l. Trees were trained to globe-shaped canopies, reaching 3-3.5 m in height and spaced at 4 × 4 m. The soil was a medium texture loam. Trees were drip irrigated with an average volume of 2100 m^3^ ha^-1^ from mid-July to the resumption of fall precipitations, typically in September.

### 2.6 Fruit growth monitoring

Fruit diameter micrometric changes were recorded at 15-min intervals with the fruit gauges described by Morandi et al. (2007a) connected to a CR-1000 datalogger (Campbell Scientific, Inc., Logan, UT, USA).

In peach, measurements were made from 25 May to 5 September on 10 fruits, one per each tree, over the entire periods of fruit development: cell division, from 25 May to 23 June; pit hardening, from 24 June to 27 July; cell expansion, from 28 July to 5 September. In ‘Keitt’ mango, diameter changes were monitored on three fruits from three different plants, over three different periods: cell division, from 27 July to 2 August; cell expansion, from 12 to 23 August; late cell expansion-maturation, from 7 to 21 September. In ‘Tommy Atkins’ mango, diameter changes were also monitored in the three fruit growth stages: from 20 to 27 July (early cell expansion), from 3 to 12 August (cell expansion), and from 24 August to 7 September (late cell expansion-maturation). In olive, measurements were carried out on one fruit per tree and 8 trees per cultivar at cell expansion (18 August to 9 September) and late cell expansion/early maturation (18 September to 18 October), as during cell division (mid-May to beginning of July) rainfall saturated the soil and canceled any possible effect of deficit irrigation. In ‘Valencia’ orange, 15 fruits (one per tree) were monitored in two periods, at the time when cell division mechanisms prevailed from 24 to 31 May (early cell division) and from 16 to 21 June (late cell division). Finally, in loquat, measurements were conducted on four fruits (one per tree) during the cell expansion and maturation stages, from 5 to 27 April. For all species, exposed fruits at about mid height of the canopy were selected.

The hourly Absolute Growth Rate (AGR; µm min^-1^) and Relative Growth Rate (RGR; µm mm^-1^ min^-1^) were calculated as follows: AGR = (D_1_ – D_0_)/(t_1_ - t_0_) and RGR = AGR/D. In the equation, D_1_ and D_0_ are the fruit diameters at time t_1_ and t_0_, respectively.

### 2.7 Climate data

In peach, climate data were retrieved from the meteorological station of Riesi (Servizio Informativo Agrometeorologico Siciliano). In mango, data of temperature and humidity were acquired with a PCEHT71 data-logger (PCE Instrument, Jupiter, FL, USA) placed in the field. In orange, climate data were acquired with two weather stations (Pessl Instruments, WZ, Austria) positioned in the experimental plot. In olive, climate data were retrieved from the meteorological station of Sciacca (Servizio Informativo Agrometeorologico Siciliano). In loquat, temperature and relative humidity were measured at one-hour intervals using an Elitech RC-51H sensor (Elitech, London, UK) placed in the farm near the experimental plot.

Data of air temperature and relative humidity were used to calculate vapor pressure deficit (VPD, kPa) using the following equation:


$$\text{VPD}={\text{VP}}_{\text{s}}–{\text{VP}}_{\text{a}},$$

Where:


$${\text{VP}}_{\text{s}}\left(\text{saturated vapor pressure}\right)=0.6108\text{ exp}\left[17.27\text{T}/\left(\text{T}+237.3\right)\right]$$

$${\text{VP}}_{\text{a}}\left(\text{actual vapor pressure}\right)=\text{RH}/100{\text{VP}}_{\text{s}}.$$

### 2.8 Statistical analysis

SYSTAT procedures (Systat Software Inc., Chicago, IL, USA) were used to carry out the daily linear regressions by group between VPD and RGR in order to obtain the coefficients (slopes) of each regression. Relationships between VPD and RGR over the whole fruit growth stages were obtained using Sigmaplot 14.0 procedures (Systat Software Inc., Chicago, IL, USA). Subsequently, the slopes of each period were compared by analysis of variance using the coefficients and standard errors from the regression output, followed by Tukey’s multiple range test (P< 0.05) when appropriate.

## 3 Results and discussion

### 3.1 Climate data

**Figure 1:**
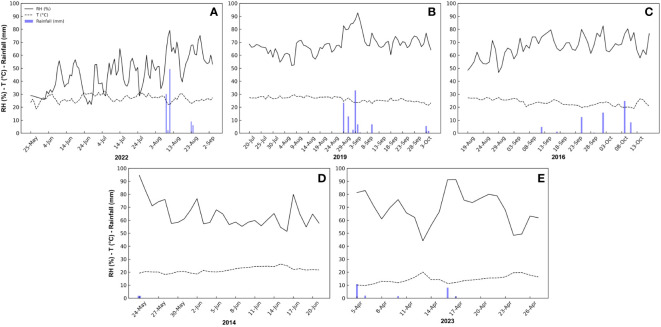
Daily trend of relative humidity (RH), temperature (T), rainfall, and vapor pressure deficit (VPD) during the monitoring periods of peach **(A)**, mango **(B)**, olive **(C)**, orange **(D)** and loquat **(E)** fruits at different locations and times in Sicily.

During peach fruit monitoring, the average temperature was 26.8°C, with a maximum daily average temperature of 32.2°C reached on 5 July, and a minimum daily average temperature of 18.6°C reached on 28 May. The average relative humidity (RH) of the period was 46.3%, with a minimum daily average value of 22.0% recorded on 28 June. The maximum daily average RH was 79.2% recorded on 11 August mainly due to a heavy rainfall event (49.4 mm). The average VPD was 2.1 kPa, with a maximum daily average value of 3.84 kPa reached on 5 July (corresponding to the maximum daily average temperature), and the minimum daily average value of 0.72 kPa on 11 August (corresponding to the maximum daily average RH and the heaviest rainfall event) (**Figure 1A**).

During mango fruit monitoring, the average temperature was 25.9°C, with a maximum daily average temperature of 35.9°C reached on 22 July, and a minimum daily average temperature of 18.0°C reached on 3 October. The average RH of the period was 68.1%, with a minimum daily average value of 32.0% recorded on 8 August. The maximum daily average RH was 98.9%, recorded on 4 September during rainfall events (6.8 mm). Consequently, the maximum VPD was recorded on 8 August (1.97 kPa) and the minimum VPD on 4 September (0.23 kPa), with an average VPD of 1.11 kPa (**Figure 1B**).

During olive fruit monitoring, the average temperature was 23.5°C, while the maximum temperature was 27.4°C, reached on 29 August, and the minimum temperature was 18.9°C, reached on 12 October. The average RH was 66.4%, with a minimum value of 46.7% recorded on 29 August and a maximum RH of 82.6% recorded on 2 October, probably because of a rainfall event (15.8 mm). The average VPD was 1.1 kPa, with a maximum value reached on 29 August (2.15 kPa) (corresponding to the maximum temperature day), and the minimum value on 25 September (0.51 kPa) (corresponding to a rainfall event of 12.4 mm) (**Figure 1C**).

During orange fruit monitoring, the average temperature was 21.7°C, with a maximum temperature of 26.2°C reached on 15 June, and a minimum temperature of 18.2°C reached on 18 May. The average RH of the period was 64.4%, with a minimum value of 51.4% recorded on 16 June and a maximum RH of 100% recorded on 24 May, probably due to a rainfall event (1.8 mm). The average VPD was 1.1 kPa, with a maximum value reached on 15 June (1.8 kPa) (corresponding to the maximum temperature day), and the minimum value on 24 may (0.1 kPa) (corresponding to the day of maximum RH) (**Figure 1D**).

During loquat fruit monitoring, the average temperature was 14.4°C, with a maximum of 20.1°C reached on 13 April, and a minimum of 9.8°C reached on 6 April. The average RH of the period was 69.3 %, with a minimum of 44.2 % reached on 13 April and a maximum of 91.3 % reached on 17 April, probably due to a rainfall event (1.5 mm). The average VPD was 0.6 kPa, with a maximum reached on 13 April (1.4 kPa) (corresponding to the day with maximum temperature and minimum RH), and the minimum on 17 April (0.1 kPa) (corresponding to the day of maximum RH) (**Figure 1E**).

### 3.2 Peach

**Figure 2:**
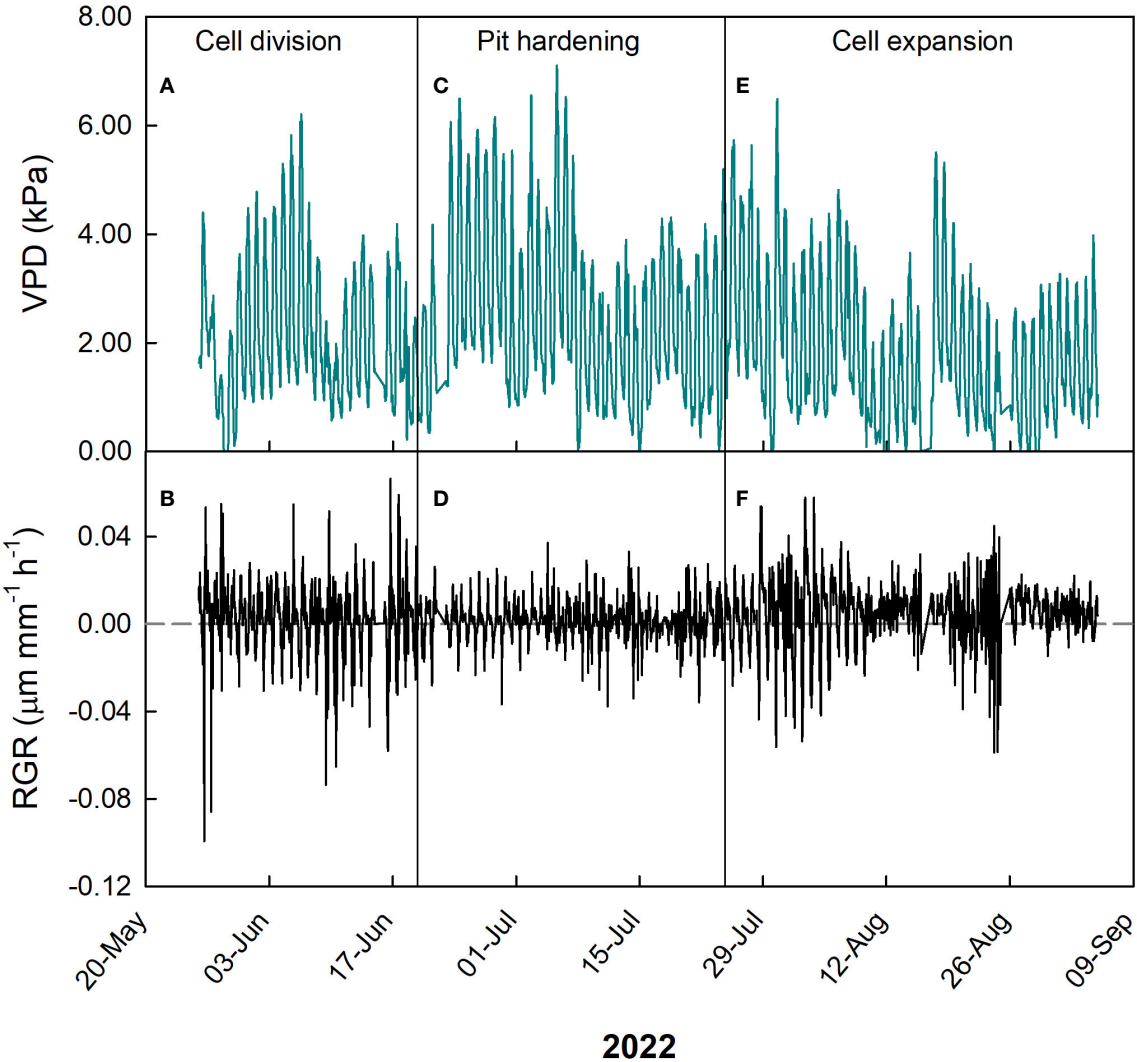
Hourly trends of vapor pressure deficit (VPD, **A**, **C**, **E**) and fruit relative growth rate (RGR, **B**, **D**, **F**) during growth stages of ‘Tardivo 2000’ peach in 2022 near Riesi, south Sicily.

During cell division, VPD ranged widely between 0 and 6.3 kPa, but the majority of measurements was between 0.5 and 2.5 kPa (**Figure 2A**), while RGR ranged between -0.04 and 0.04 µm mm^-1^ min^-1^, with most values concentrated between 0.02 and -0.02 µm mm^-1^ min^-1^ (**Figure 2B**).

**Figure 3:**
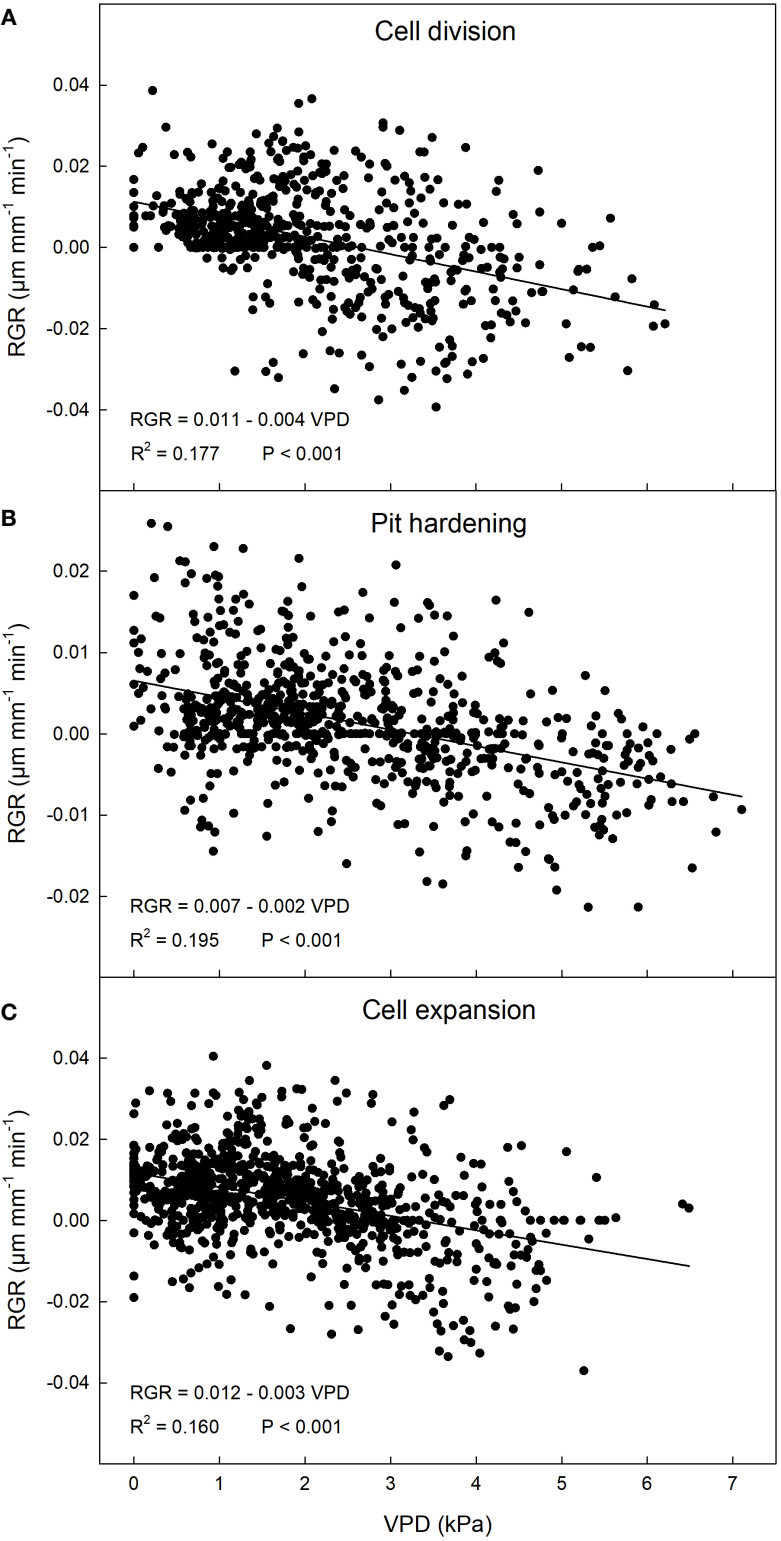
Relationship between vapor pressure deficit (VPD) and relative growth rate (RGR) in ‘Tardivo 2000’ peach at cell division **(A)**, pit hardening **(B)** and cell expansion **(C)** in 2022 near Riesi, south Sicily.

A significant weak negative linear relationship between VPD and RGR was found at this stage (**Figure 3A**). Under the environmental conditions of the site and the period of the year, an inverse relationship may be expected since high levels of VPD may induce stomatal closure, resulting in reduced photosynthesis and fruit growth (Guichard et al., 2005).

A negative linear relationship between these two parameters was detected also at pit hardening (**Figure 3B**). At this stage, RGR ranged between -0.022 and 0.026 µm mm^-1^ min^-1^ but most of the values were in the range between -0.005 and 0.01 µm mm^-1^ min^-1^, while VPD ranged widely between 0 and 7.2 kPa with most values between 1 and 4 kPa (**Figure 2**). As expected, in this period fluctuations in fruit RGR were smaller than at cell division (Lo Bianco and Scalisi, 2019; Scalisi et al., 2019a).

At cell expansion, RGR ranged between -0.04 and 0.04 µm mm^-1^ min^-1^ but most of the values were included between 0 and 0.02 µm mm^-1^ min^-1^. VPD ranged between 0 and 6.7 kPa, with most of the values concentrated between 0 and 3.5 kPa (**Figure 2**). At this stage, a negative linear relationship between VPD and RGR similar to the previous two stages was detected (**Figure 3C**). The inverse relationship between VPD (one of the parameters driving transpiration) and fruit RGR at this stage could be attributed to changes in leaf conductance and their consequent ability to absorb water along the day, as well as to the competition for water between leaves and fruit (Grilo et al., 2019). It is worth noting that a significant portion of xylem water is directed towards transpiring leaves in the morning and during the midday, while fruit xylem inflow and RGR remain relatively low, as evidenced in peach (Morandi et al., 2007b), apple (Lang, 1990) and kiwifruit (Morandi et al., 2006). During this daytime, leaves act as strong water sinks (primarily influenced by VPD), and there is even a possibility of water loss by the fruit through backflow (Constantinescu et al., 2020). This causes a reduction in fruit growth. Large fluctuations in fruit RGR are the result of partial dehydration of the plants in part due to high VPD values (**Figure 2B**), in line with previous observations (Scalisi et al., 2019a).

Surprisingly, the dependence of RGR on VPD at cell expansion, when fruit water content is highest and cell growth strongly depends on water-driven cell turgor, was similar to the one at cell division, when water content is less directly involved in fruit growth. This suggests that similar levels of leaf stomatal limitation at the two fruit growth stages (cell division and cell expansion) may generate similar fruit growth regulation operated through totally different mechanisms, carbon fixation at cell division and water flows at cell expansion. This ultimately generates similar responses of fruit growth to atmospheric conditions. These results agree with results obtained by Morandi et al. (2007b), who found that patterns of daily fruit growth and phloem, xylem and transpiration flows are similar at cell division and expansion stages. Moreover, strong and similar linear relationships between fruit transpiration rate and VPD were found at both cell division and expansion.

**Figure 4:**
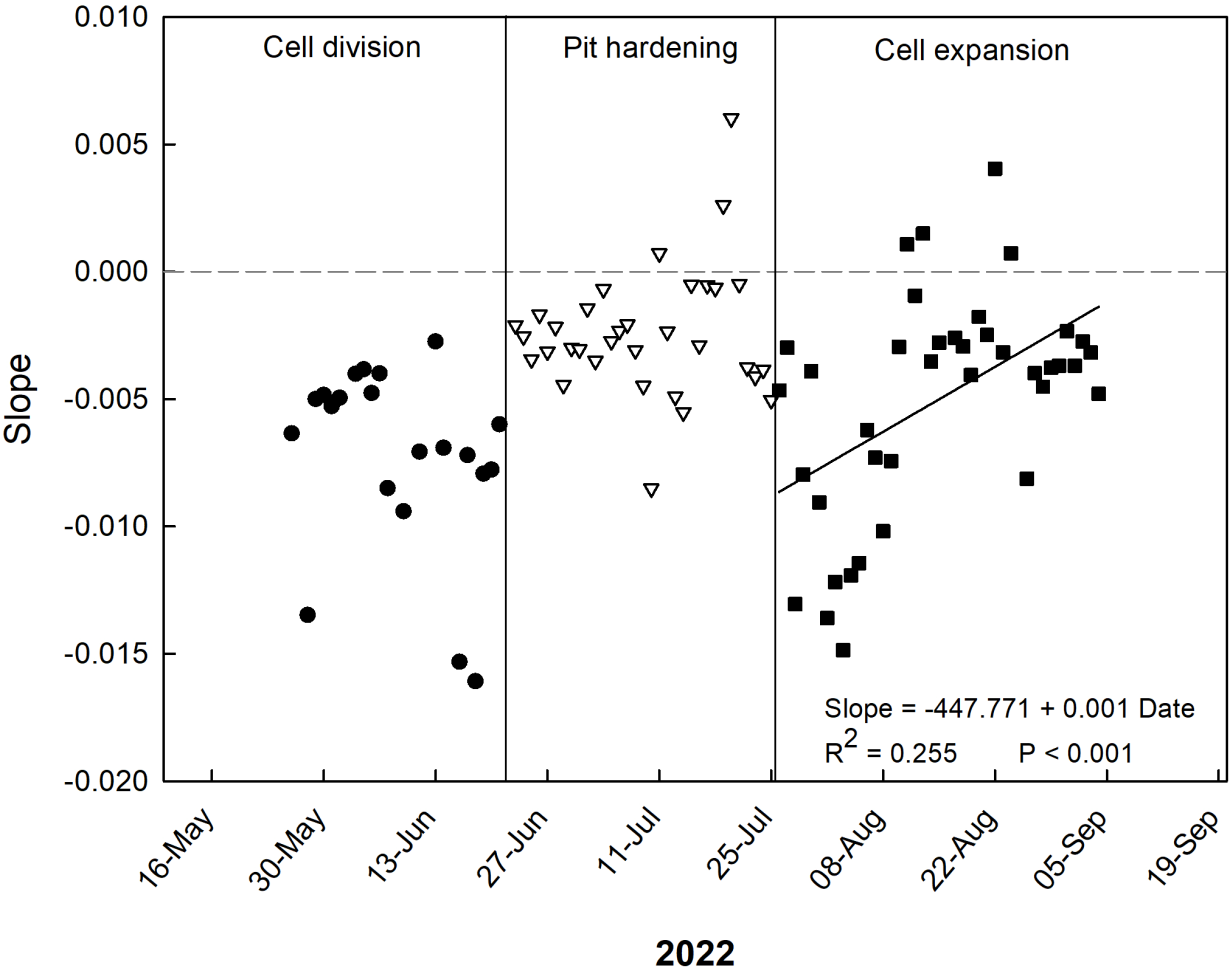
Trends of coefficients (slopes) of the daily linear regressions between VPD and RGR at the three stages of ‘Tardivo 2000’ peach fruit growth in 2022 near Riesi, south Sicily.

Regarding the slopes of the daily relationships between VPD and RGR, only negative values can be seen during the period from late May to mid-July (**Figure 4**). Very negative values were found on June 14 and 15 (cell division stage), in which fruit growth was strongly influenced by VPD (**Figure 4**). In mid/late July, at the end of the pit hardening phase, a peak of positive values was found, indicating a period of optimum plant hydration as a result of frequent and abundant irrigation events. During cell expansion stage, a general linear increase of the slopes of the daily VPD-to-RGR relationships was found, indicating a transition from inverse to no relationship between VPD and RGR, i.e. a loss of fruit sensitivity to the atmospheric conditions.

### 3.3 Mango

**Figure 5:**
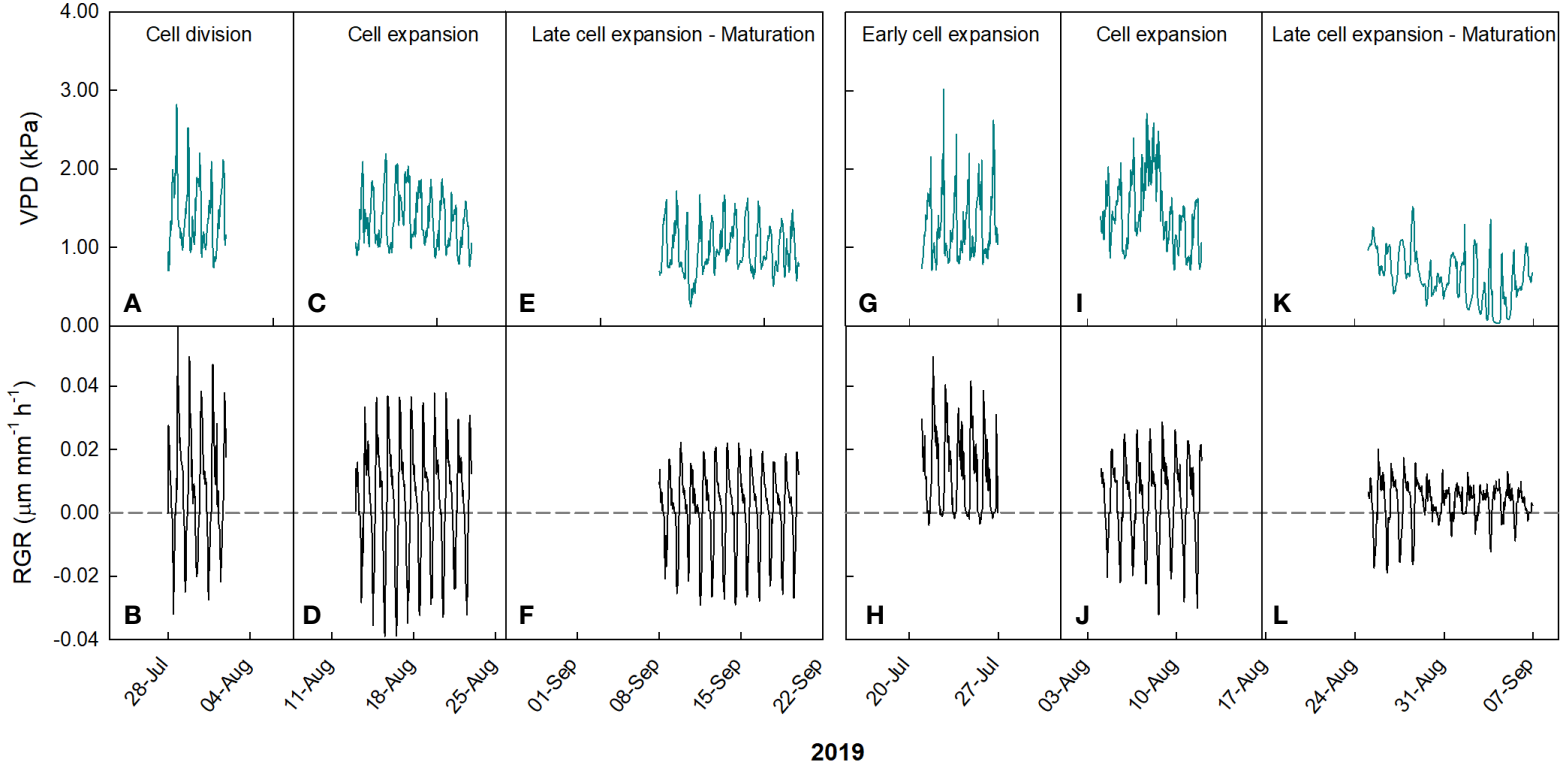
Hourly trends of vapor pressure deficit (VPD) in ‘Keitt’ **(A, C, E)** and ‘Tommy Atkins’ **(G, I, K)** mango and fruit relative growth rate (RGR) in ‘Keitt’ **(B, D, F)** and ‘Tommy Atkins’ **(H, J, L)** during growth stages in 2019 near Caronia, northeast Sicily.

In ‘Keitt’, during the cell division stage, RGR values ranged from -0.03 to 0.06 µm mm^-1^ min^-1^, and VPD from 0.71 to 2.81 kPa (**Figure 5**).

**Figure 6:**
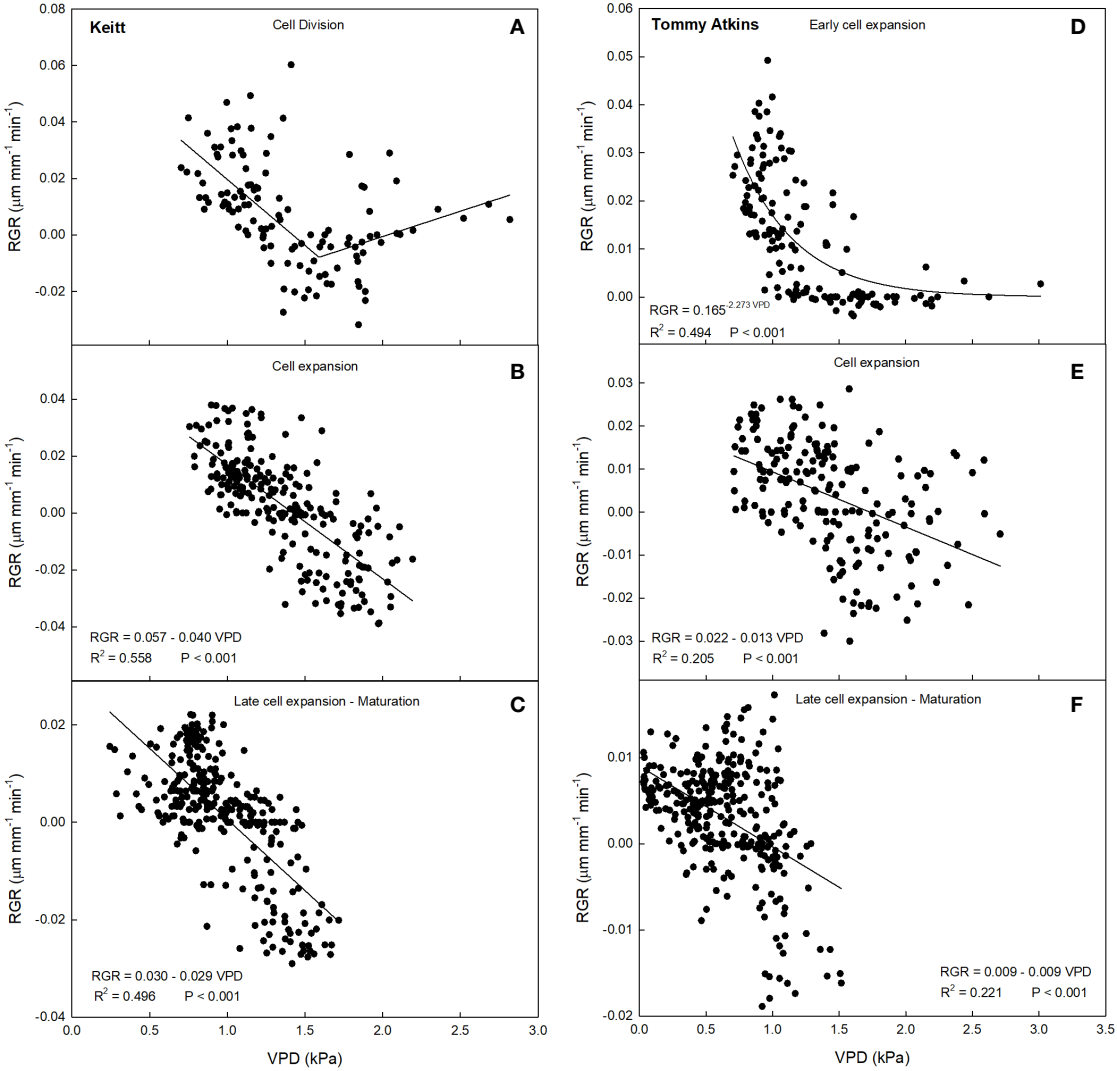
Relationship between vapor pressure deficit (VPD) and relative growth rate (RGR) in ‘Keitt’ **(A–C)** and ‘Tommy Atkins’ **(D–F)** mango during different fruit growth stages in 2019 near Caronia, northeast Sicily.


A 2-segment piecewise linear relationship was found during the cell division stage (R^2^ = 0.36, P< 0.001), with the following system (**Figure 6A**):

$$RGR=\{\begin{array}{c} \frac{0.033\left(1.591-VPD\right)-0.008\left(VPD-VPD_{min}\right)}{1.591-VPD_{min}},VPD\leq 1.591kPa\\ \frac{-0.008\left(VPD_{max}-VPD\right)+0.014\left(VPD-1.591\right)}{VPD_{max}-1.591},VPD>1.591kPa \end{array}$$

The first segment of the piecewise has a negative slope, while the second has a positive slope. The breakpoint corresponds to a VPD value of 1.591 kPa. This means that the fruit, beyond the breakpoint value, increased its growth rate along with increasing VPD. The points after the breakpoint corresponded to times from 17:00 to 19:00, so when the fruits were regaining turgidity. This likely occurred because beyond that value, the plant tended to favor fruit growth instead of leaf carbon and water accumulation in the afternoon hours. A similar behavior was observed by Leonardi et al. (2000) in tomato fruit, where around 17:00 the fruit increased its growth rate despite high VPD values, but no explanation was provided by the Authors. At cell expansion stage, RGR values ranged from -0.04 to 0.04 µm mm^-1^ min^-1^, and VPD from 0.74 to 2.19 kPa (**Figure 5A**). In this case, a negative linear relationship between VPD and RGR was detected (R^2^ = 0.56, P< 0.001) (**Figure 6B**). When cell expansion mechanisms prevail, water exchanges between the fruit and the atmosphere or the rest of the tree are main drivers of fruit growth, i.e., cell expansion is strongly influenced by the daily fluctuations of VPD. In addition, an increase in VPD, often caused by high temperatures, results in an increase in transpiration, which in turn reduces the xylem water potential and consequently decreases the xylem flow to the fruit, slowing down its enlargement (Tombesi et al., 2014). During the late cell expansion-maturation stage, RGR values ranged from -0.03 to 0.02 µm mm^-1^ min^-^1, and VPD from 0.23 to 1.71 kPa (**Figure 5A**). Also at this stage, a negative linear relationship between the two parameters was identified (**Figure 6C**). However, the slope of the regression line at this stage is significantly less negative than the one at the cell expansion stage (P< 0.05). This indicates that the fruit, as maturation approaches, becomes less dependent on atmospheric environment probably due to both stomatal and xylem isolation mechanisms that prevent fruit water loss, while water inflow still occurs via the phloem. Xylem interruption mechanisms near maturation have been documented in kiwifruit (Montanaro et al., 2012), grape (Keller et al., 2006; Choat et al., 2009), sweet cherry (Grimm et al., 2017) and apple (Dražeta et al., 2004).

**Figure 7:**
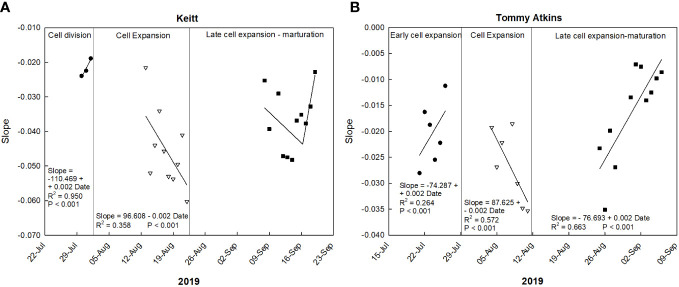
Trends of coefficients (slopes) of the daily linear regressions between VPD and RGR at different growth stages of ‘Keitt’ **(A)** and ‘Tommy Atkins’ **(B)** mango fruit in 2019 near Caronia, northeast Sicily.


Such responses are confirmed by the trend of the daily slopes of the relationships between VPD and RGR during the entire monitoring period (**Figure 7A**). At the cell division and late cell expansion-maturation stages, fruit growth was less influenced by VPD compared to the cell expansion stage, where coefficients were generally more negative, and an inverse trend was detected. This is in line with the principle that, at this stage, fruit growth is directly linked to water relations and xylem functionality, and thereby it responds quickly to VPD changes. Hence, going forward along this stage, the most negative slope values were reached. In the late cell expansion-maturation stage, the trend of daily slopes follows a piecewise pattern. Initially fruit growth responded to changes in VPD similarly to cell expansion. At this specific point, climacteric fruits are characterized by a sharp increase of respiration, which is highly influenced by VPD and has been found to account for up to 39% of water losses in pear fruits (Xanthopoulos et al., 2017). However, a breakpoint is reached on 14 September (43722; days since 1 January 1900) at RGR = -0.044, and after that the fruit tended to respond less and less to VPD (**Figure 7A**). The piecewise model was the following:

$$Slope=\{\begin{array}{c} \frac{-0.033\left(43722.306-Date\right)-0.044\left(Date-Date_{min}\right)}{43722.306-Date_{min}},Date\leq 43722.306\\ \frac{-0.044\left(Date_{max}-Date\right)-0.024\left(Date-43722.306\right)}{Date_{max}-43722.306},VPD>43722.306 \end{array}$$

This may be because during maturation xylem flows and functionality are reduced and the fruit depends more on the phloem functionality and thereby on the vacuole osmotic gradient (Nordey et al., 2015). Moreover, it could be that at maturation, there is a threshold of water entering to the fruit, and the rest of water is recycled by backflow through xylem as observed in grapes (Keller et al., 2015). In ‘Tommy Atkins’, during the early cell expansion stage, RGR ranged from 0 to 0.05 µm mm^-1^ min^-1^, and VPD from 0.70 to 3.01 kPa (**Figure 5B**). At this stage, the relationship between VPD and RGR was best described by an exponential decay model (**Figure 6D**). The model shows a strong inverse relation at the VPD interval 0.7 to 1.2 kPa, becoming gradually weaker until a VPD of about 1.5 kPa after which the effect on RGR is lost. At full cell expansion stage, RGR values ranged from -0.03 to 0.02 µm mm^-1^ min^-1^ and VPD from 0.71 to 2.70 kPa (**Figure 5B**). At this stage, a negative linear relationship between VPD and RGR was detected (**Figure 6E**), like in ‘Keitt’. Finally, in the transition period between late cell expansion and maturation, RGR values ranged from -0.02 to 0.02 µm mm^-1^ min^-1^ and VPD from 0.01 to 1.52 kPa. A negative linear relationship was also found at this stage (**Figure 6F**), but with a significantly lower slope of the regression line compared to the cell expansion stage (P< 0.05). This difference confirms the trends observed in ‘Keitt’ fruit, i.e., the fruit tends to be decreasingly dependent on the atmospheric environment as maturation approaches.

Also in ‘Tommy Atkins’, these responses are confirmed by the trend of the daily slopes of the relationships between VPD and RGR during the entire monitoring period (**Figure 7B**). The strongest effect of VPD on fruit growth appears to be just in the middle of the cell expansion phase (end of July-beginning of August), while it tends to disappear after 2 September, when maturation processes begin.

### 3.4 Olive

**Figure 8:**
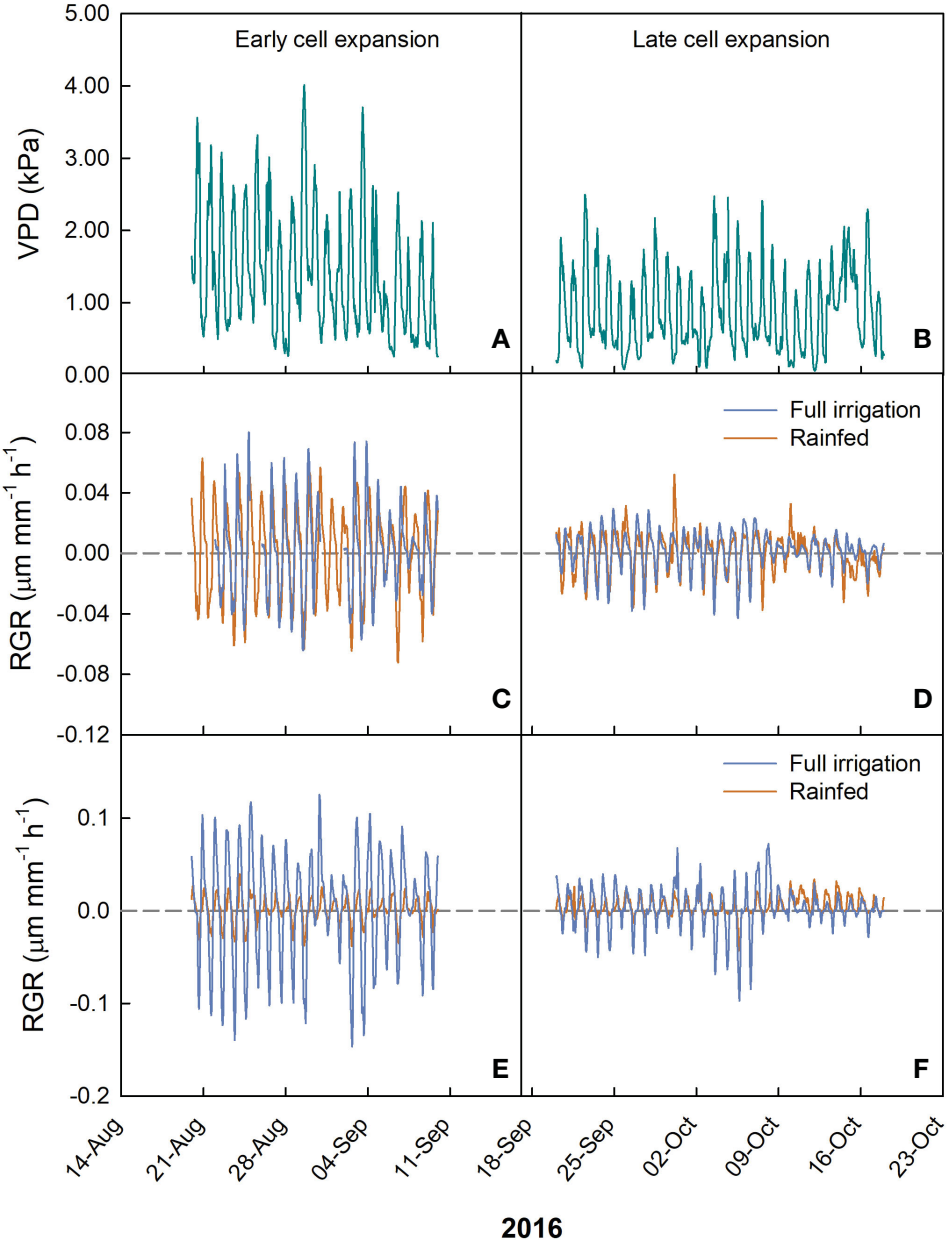
Hourly trends of vapor pressure deficit (VPD; **A, D**) and fruit relative growth rate (RGR) during early and late cell expansion stages of fruit growth in ‘Nocellara del Belice’ **(B, E)** and ‘Olivo di Mandanici’ **(C, F)** olive under full irrigation (blue lines) and rainfed (orange lines) conditions in 2016 near Sciacca, in southwestern Sicily.

In olive, two cultivars (Nocellara del Belice and Olivo di Mandanici) were considered at two different irrigation levels and at two fruit growth stages, cell expansion and late cell expansion-maturation. At the early cell expansion stage, the range of VPD was the same in both cultivars and irrigation treatments, from 0.02 to 4.01 kPa (**Figure 8A**). In ‘Nocellara del Belice’, RGR varied from -0.04 to 0.05 µm mm^-1^ min^-1^ in rainfed trees, and -0.06 to 0.08 µm mm^-1^ min^-1^ in full irrigated trees (**Figure 8B**), showing a relatively small but significant negative effect of water deficit on fruit growth. In ‘Olivo di Mandanici’, the range of RGR during early cell expansion was -0.04 to 0.04 µm mm^-1^ min^-1^ in fruits of rainfed trees and -0.14 to 0.12 µm mm^-1^ min^-1^ in fruits of full irrigated trees (**Figure 8C**), showing a relatively bigger effect of water deficit on fruit growth of this cultivar compared to Nocellara del Belice.

At late cell expansion-maturation stage, the range of VPD for both cultivars and irrigation treatments was from 0.04 to 2.46 kPa (**Figure 8D**). In ‘Nocellara del Belice’ fruit, the range of RGR was -0.04 to 0.03 µm mm^-1^ min^-1^ in full irrigated trees, and -0.04 to 0.05 µm mm^-1^ min^-1^ in rainfed trees (**Figure 8E**), showing no effect of water deficit on fruit growth. In ‘Olivo di Mandanici’ fruit, RGR ranged from -0.09 to 0.07 µm mm^-1^ min^-1^ in full irrigated trees and from -0.04 to 0.04 µm mm^-1^ min^-1^ in rainfed trees (**Figure 8F**), showing again major reductions of fruit growth in response to water deficit.

**Figure 9:**
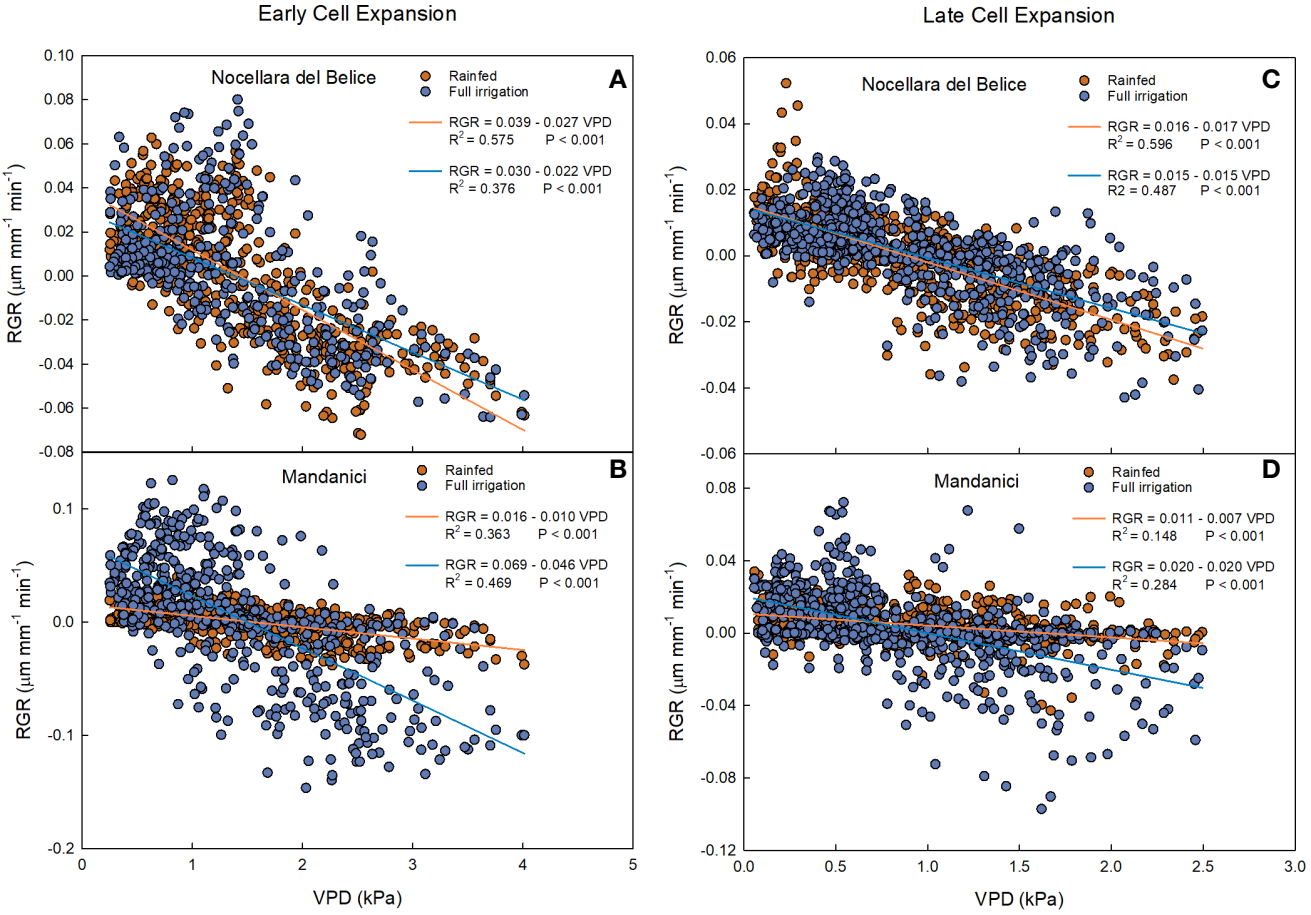
Relationship between vapor pressure deficit (VPD) and fruit relative growth rate (RGR) at early cell expansion (left) and late cell expansion (right) stages in ‘Nocellara del Belice’ **(A, C)** and ‘Olivo di Mandanici’ **(B, D)** olive trees under full irrigation (blue) and rainfed (orange) conditions in 2016 near Sciacca, in southwestern Sicily.

At the early cell expansion stage, a negative linear relationship was found between RGR and VPD in both fruits from rainfed and full irrigated trees (**Figure 9A**). Yet, the slope of the regression line from rainfed trees was more negative than the one from full irrigated trees (P< 0.001) during the early cell division. This indicates that at this stage, the negative effect of VPD on fruit RGR of ‘Nocellara del Belice’ is greater under non-optimal water conditions than under full irrigation, suggesting that irrigation compensates the fruit growth reduction due to VPD.

Negative linear relationships between RGR and VPD were found in both rainfed and full irrigated ‘Olivo di Mandanici’ trees (**Figure 9B**). In this case, the slope of full irrigated trees was significantly more negative than that of rainfed trees (P< 0.001), which was barely different from 0. This suggests that fruits of ‘Olivo di Mandanici’ trees under water deficit are able to keep adequate hydration by reducing growth and not responding to VPD changes. This may be accomplished by accumulating organic solutes and lowering fruit osmotic potential to maintain good hydration levels under unfavorable tree water status, indicating a greater ability of ‘Olivo di Mandanici’ to adjust osmotically, and a lower degree of isohydricity, compared to ‘Nocellara del Belice’ (Lo Bianco et al., 2013; Lo Bianco and Scalisi, 2017). It may be assumed that the relationship between RGR and VPD is strictly related to the ability of the plant to tolerate water deficit: the more tolerant the plant is, the less the fruit will be affected by VPD. This is in line with results found by Scalisi et al. (2020) indicating that ‘Olivo di Mandanici’ is more tolerant to water deficit than ‘Nocellara del Belice’, i.e. an evident genetic component.

Also at late cell expansion-maturation stage, negative linear relationships were found between VPD and RGR in rainfed and in full irrigated ‘Nocellara del Belice’ trees (**Figure 9C**). Similar to the early cell expansion stage, the slope of the regression line from rainfed trees was more negative than the one from full irrigated trees (P = 0.013). At both stages, ‘Nocellara del Belice’ showed the tendency of not reducing fruit growth under water deficit, probably at the expenses of tree water status.

At this stage, in ‘Olivo di Mandanici’, weaker negative linear relationships (in terms of slope and R^2^) (**Figure 9D**) between VPD and RGR were found in both irrigation treatments compared to the early cell expansion stage (**Figure 6D**). This happened probably because the fruit of ‘Olivo di Mandanici’ was getting close to maturity and tended to isolate itself from environmental factors (VPD), so the fruit response to VPD changes was decreasing. Opposite to what happened in ‘Nocellara del Belice’, the slope of the regression line of full irrigated ‘Olivo di Mandanici’ trees was significantly more negative than that of rainfed trees (P< 0.001). This outcome can be related to the previous information: when a plant (such as the ‘Olivo di Mandanici’) shows greater tolerance to water stress, it suffers less impact on fruit growth from VPD.

**Figure 10:**
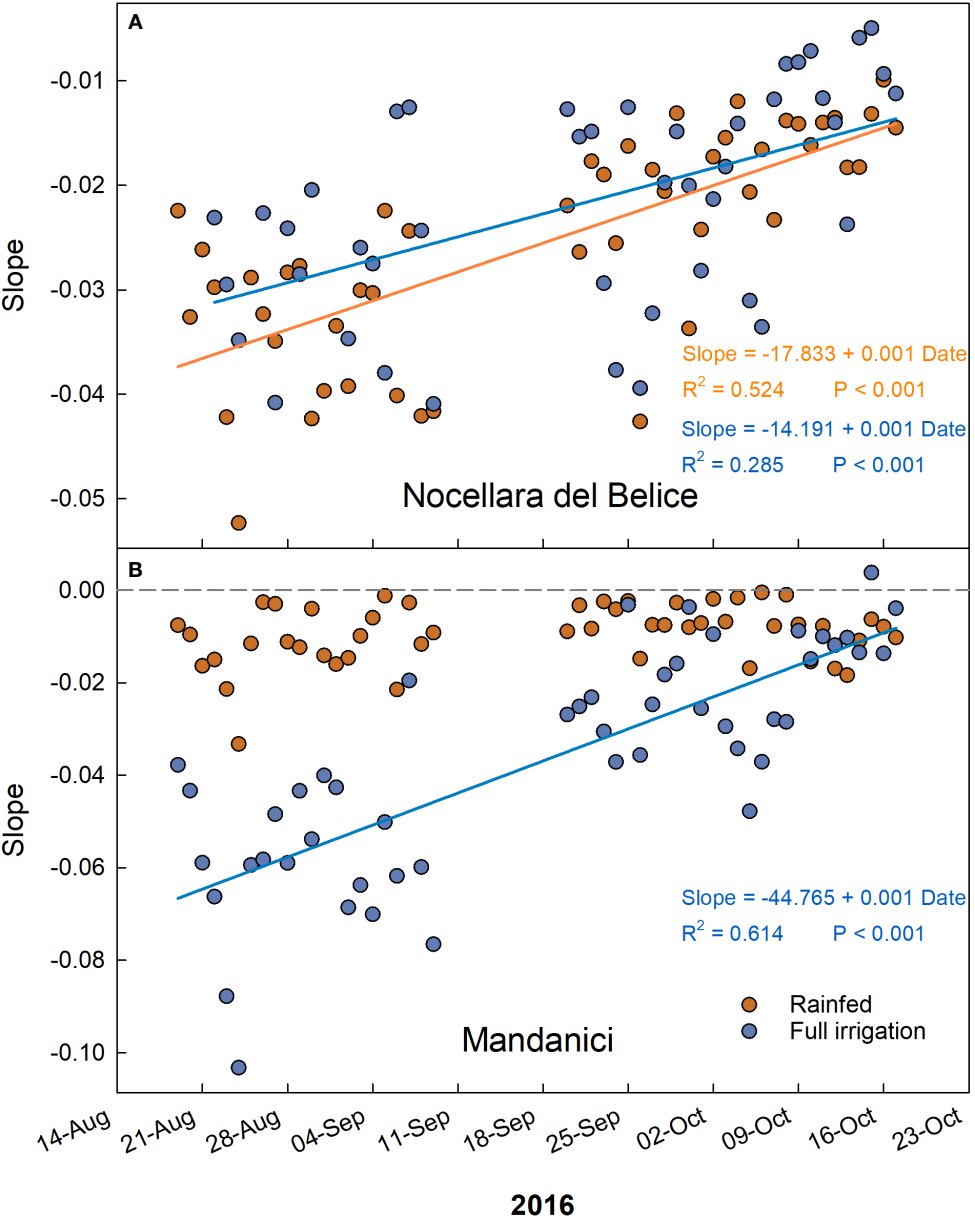
Trends of coefficients (slopes) of the daily linear regressions between VPD and RGR across early to late cell expansion stages of ‘Nocellara del Belice’ **(A)** and ‘Olivo di Mandanici’ **(B)** olive fruit growth in 2016 near Sciacca, in southwestern Sicily.

The trends of the daily slopes of the relationships between VPD and RGR during the entire monitoring period showed a linear increase in all cases but in ‘Olivo di Mandanici’ under water deficit (**Figure 10**). In this last case, slopes remained constantly high, near 0 (**Figure 10B**), indicating a general lack of effect of VPD on fruit growth. The increasing trends confirm that the effect of VPD on fruit growth at this stage is linked to cell expansion activity. Indeed, cell expansion activity is directly associated to fruit water relations and therefore influenced by daily fluctuations in temperature, relative humidity, and vapor pressure deficit (VPD) (Lescourret et al., 2001).

### 3.5 Orange

**Figure 11:**
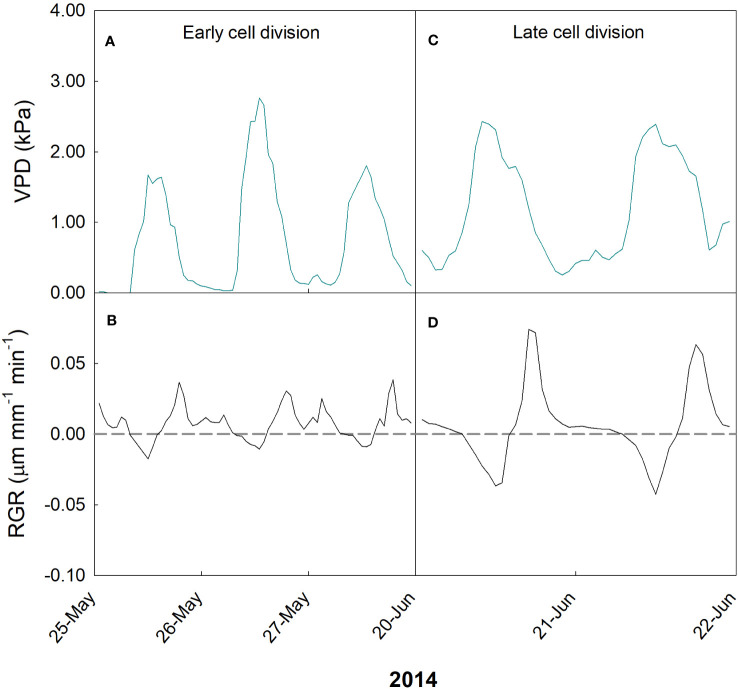
Hourly trends of vapor pressure deficit (VPD; **A, C**) and fruit relative growth rate (RGR; **B, D**) during early **(A, B)** and late **(C, D)** cell division stages of fruit growth in ‘Valencia’ orange in 2014 at the University of Palermo, Sicily.

In the case of ‘Valencia’ orange, two periods were monitored at the time when cell division mechanisms prevailed: before harvest of previous season fruit (24-31 May 2014) and after their harvest (16-21 June 2014). In May, VPD ranged from 0 (corresponding to rainy days) to 2.8 kPa (**Figure 11A**), while RGR ranged from -0.02 to 0.04 µm mm^-1^ min^-1^ (**Figure 11B**). On the other hand, in June, VPD ranged from 0.2 to 2.5 kPa (**Figure 11C**), and the fruit showed a wider range of RGR, from -0.04 to 0.08 µm mm^-1^ min^-1^ (**Figure 11D**).

**Figure 12:**
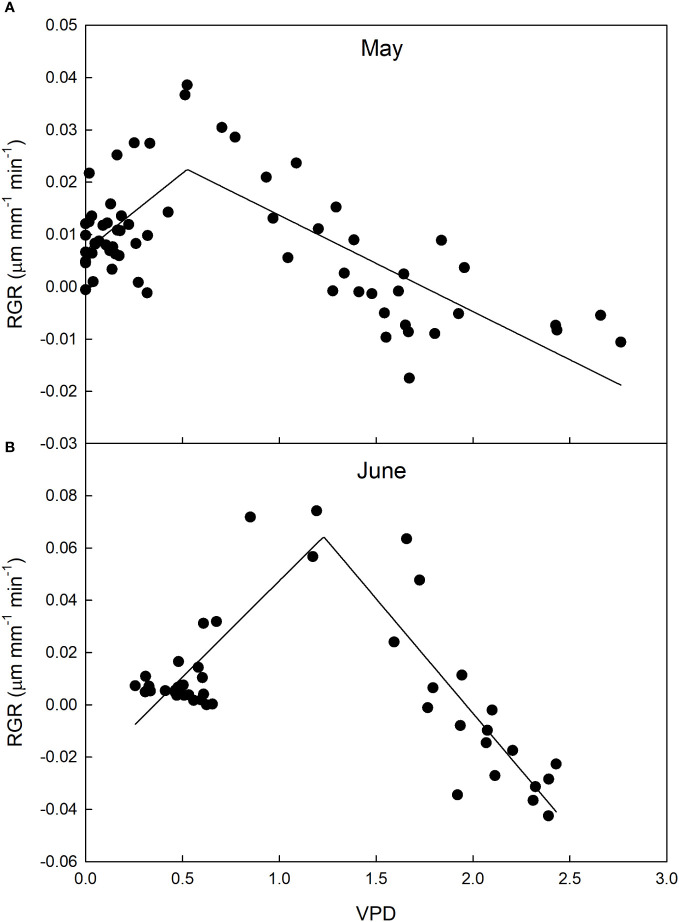
Relationship between vapor pressure deficit (VPD) and relative growth rate (RGR) of ‘Valencia’ orange fruit at cell division stage in May **(A)** and June **(B)**, before and after harvest of mature fruit, respectively in 2014 at the University of Palermo, Sicily.


Fruit growth dynamics were significantly influenced by VPD in both measurement periods. Specifically, a two-segment piece-wise model was found that accurately describes the relationship between VPD and fruit RGR (**Figure 12**). In May, the piecewise system was the following:

$$RGR=\{\begin{array}{c} \frac{0.007\left(0.524-VPD\right)+0.022\left(VPD-VPD_{min}\right)}{0.524-VPD_{min}},VPD\leq 0.524kPa\\ \frac{0.022\left(VPD_{max}-VPD\right)-0.019\left(VPD-0.524\right)}{VPD_{max}-0.524},VPD>0.524kPa \end{array}$$

In June, the piecewise system was the following:


$$RGR=\{\begin{array}{c} \frac{-0.007\left(1.229-VPD\right)+0.064\left(VPD-VPD_{min}\right)}{1.229-VPD_{min}},VPD\leq 1.229kPa\\ \frac{0.064\left(VPD_{max}-VPD\right)-0.041\left(VPD-1.229\right)}{VPD_{max}-1.229},VPD>1.229kPa \end{array}$$

At lower VPD values (during the night and early morning), there was a direct linear relationship between VPD and RGR, whereas an inverse relationship was observed at high VPD levels during the day and evening. These trends were consistent for both May and June, with a significant (P< 0.001) difference in the breakpoints, where the relationship inverted its trend. The inverse relationship between VPD and RGR shown in the second segment of the piecewise regression is similar to that observed in other fruits (see above) and can be explained by the negative effect of high VPD levels on stomatal conductance, which reduces fruit growth by limiting carbon assimilation. During the evening, there is typically a reduction in leaf transpiration (Matos et al., 1998; Morandi et al., 2014), and consequently stem sap flow also decreases, in accordance with VPD. This period is commonly when fruits initiate rehydration through xylem transportation (Morandi et al., 2010; Morandi et al., 2014), leading to an increase in RGR. In our study, this resulted in an inverse relationship between RGR and VPD. A breakpoint at higher VPD in June than in May can be explained by the removal of older fruits competing for carbon and water with young fruitlets (Grilo et al., 2019).

In the case of ‘Valencia’ orange, no specific trend of the coefficients (slopes) of the daily linear regressions between VPD and RGR was found, most likely because the two monitored periods were close in time and part of the same fruit development stage, early vs late cell division.

### 3.6 Loquat

**Figure 13:**
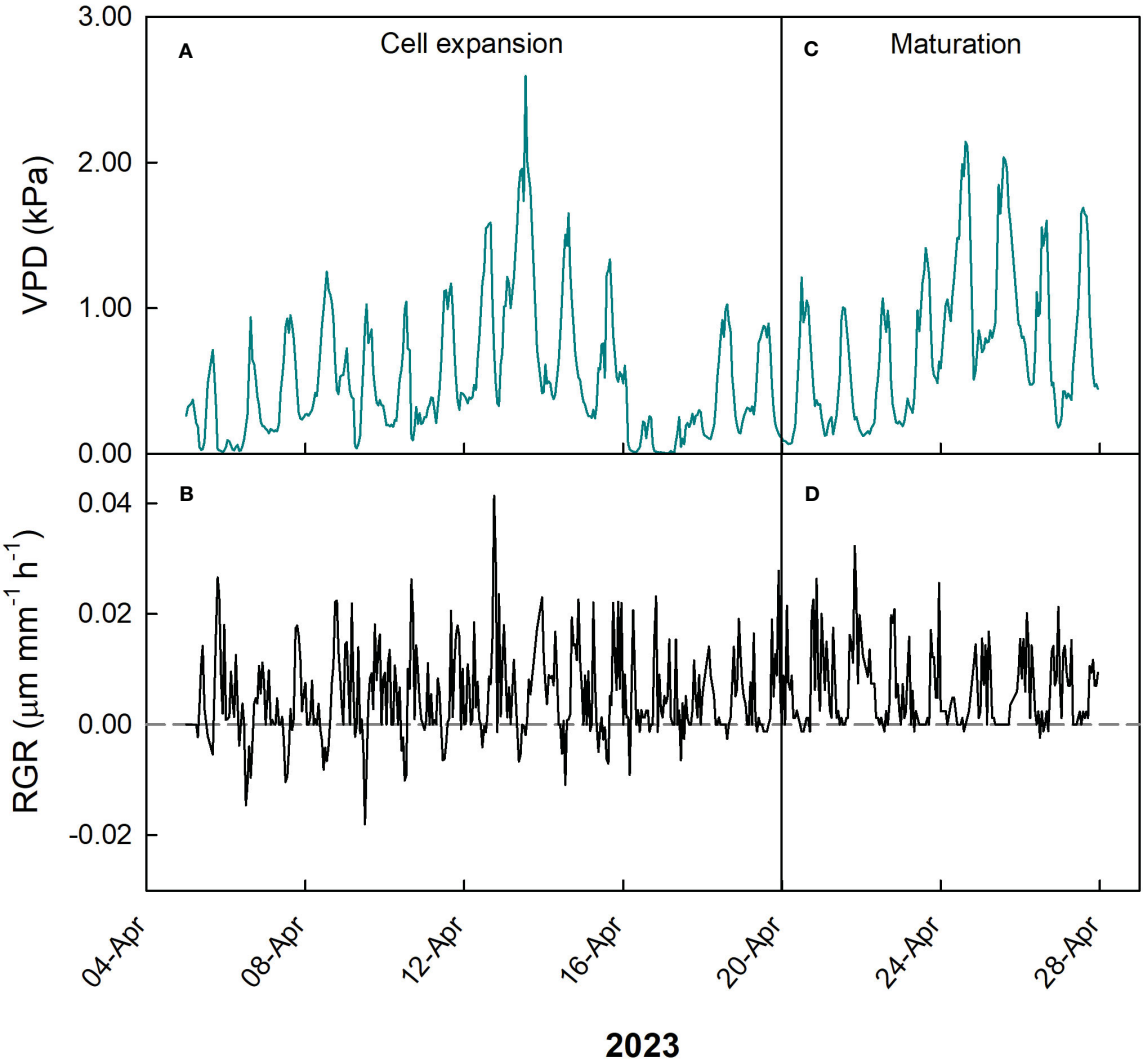
Hourly trends of vapor pressure deficit (VPD; **A, C**) and fruit relative growth rate (RGR; **B, D**) during cell expansion **(A, B)** and maturation **(C, D)** stages of fruit growth in ‘Nespolone di Trabia’ loquat in 2023 in Ciaculli, near Palermo, Sicily.

During cell expansion stage of loquat fruit growth, VPD ranged between 0 and 2.6 kPa, but the majority of measurements was between 0.5 and 1.2 kPa (**Figure 13A**), while RGR ranged between -0.02 and 0.04 µm mm^-1^ min^-1^, with most values concentrated between -0.005 and 0.02 µm mm^-1^ min^-1^ (**Figure 13B**). At the fruit maturation stage, VPD ranged between 0.07 and 2.1 kPa (**Figure 13C**), while RGR ranged between -0.002 and 0.03 µm mm^-1^ min^-1^ (**Figure 13D**), showing a relatively lower fluctuation of RGR compared to cell expansion stage. The latter is consistent with the beginning of fruit maturation.

**Figure 14:**
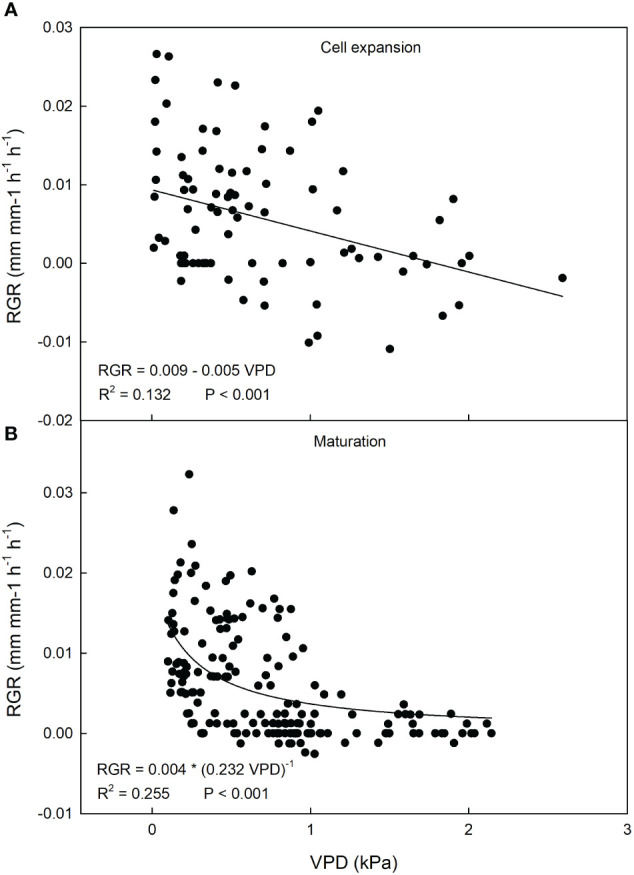
Relationship between vapor pressure deficit (VPD) and relative growth rate (RGR) of ‘Nespolone di Trabia’ loquat fruit at cell expansion **(A)** and maturation stage **(B)** in 2023 in Ciaculli, near Palermo, Sicily.

At cell expansion stage, a weak negative linear relationship between VPD and RGR was found (**Figure 14A**). Considering the relatively mild VPD conditions due to the period of the year, a weak effect on fruit growth can be expected. At fruit maturation, a negative hyperbolic decay model best described the relationship between VPD and RGR (**Figure 14B**). Just like in olive and mango, this indicates that, as maturation progresses, the fruit becomes less dependent on atmospheric environment probably due to both the lowering of fruit osmotic potential and xylem isolation mechanisms that prevent fruit water loss.

**Figure 15:**
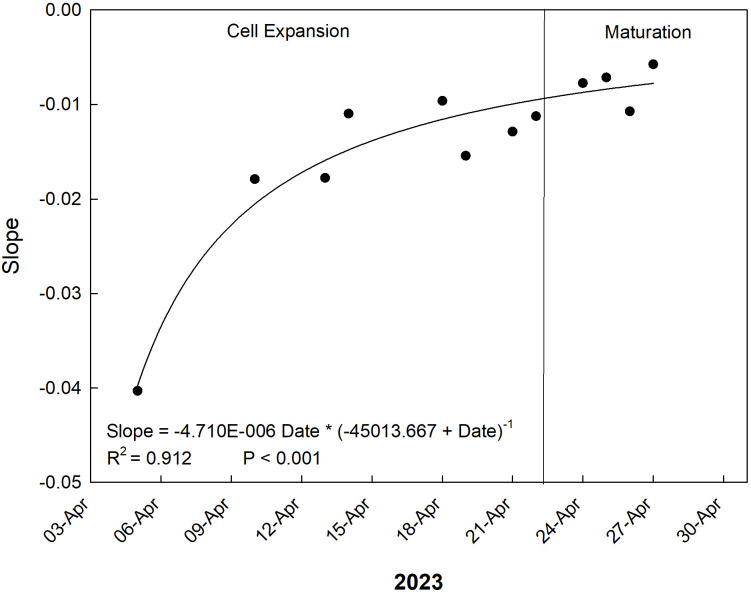
Trends of coefficients (slopes) of the daily linear regressions between VPD and RGR across late cell expansion to maturation stages of ‘Nespolone di Trabia’ loquat in 2023 in Ciaculli, near Palermo, Sicily.

As for the slopes of the daily relationships between RGR and VPD, a hyperbolic trend tending to 0 (non-significant relationship) was observed (**Figure 15**). In other words, as the fruit goes from cell expansion stage to maturation, its response to changes in VPD becomes weaker and weaker until it reaches a quasi-steady state of no response during maturation. Our results are in line with previous observations showing that, starting at veraison and all throughout maturation, the growth rate of the fruit is not particularly affected by climatic factors (Gariglio et al., 2002). The maturation period is indeed mainly characterized by decreasing acidity, sugar accumulation, color development, softening of the pulp tissue, and a rapid increase in the fresh weight of the pulp tissue (Lin et al., 1999).

## 4 Conclusions

Overall, data collected at different growth stages of peach, mango, olive, orange and loquat fruits indicate a general effect of atmospheric water demand on fruit growth. Specifically, a consistent and more marked negative relationship between VPD and RGR was observed at cell expansion stage, when fruit growth is directly depending on water intake driving cell turgor. This indicates that, regardless of fruit type, VPD could be a powerful indicator of fruit growth and a useful parameter to be monitored for tree water management at this stage. Another behavior common to all observed species (with climacteric and non-climacteric fruits) was the gradual loss of relationship between VPD and RGR at the onset of fruit maturation. At this stage, fruit growth in size is generally programmed to stop and little to no effect of ambient conditions on RGR is observed, either because fruits tend to get hydraulically isolated from the outside and the rest of the plant or because excess of water entering the fruit may be recycled by backflow through the xylem. Sensitivity of fruit growth to ambient conditions at the fruit cell division stage seems to vary with species, time, and probably soil and atmospheric water deficit. We only have data from peach, mango, and orange and they do not seem to express a common behavior or mechanism. Especially ‘Keitt’ mango and ‘Valencia’ orange fruit growth responded to VPD in opposite ways; of course, time of the year and VPD levels were very different in the monitoring periods of the two species. At pit hardening stage of peach fruit growth, a relatively weak relationship was observed between VPD and RGR, and this is not surprising as fruit growth in size at this stage slows down significantly masking off any effect of atmospheric water demand on fruit growth. Finally, according to our findings, we can say that VPD may be a useful indicator of fruit growth and tree irrigation needs mainly when the cell expansion process prevails during fruit growth. These results are important especially considering the global change scenario predicting more tropical nights with higher temperatures and relative humidity but also temperature and drought extremes during summer days. Collection of more data at the cell division stage from fruits of different species might serve to clarify the role of atmospheric water demand on fruit growth and the possible usefulness of VPD as an indicator of tree irrigation needs.

## Data availability statement

The raw data supporting the conclusions of this article will be made available by the authors, without undue reservation.

## Author contributions

RB: Conceptualization, Data curation, Formal analysis, Funding acquisition, Methodology, Project administration, Supervision, Writing – original draft, Writing – review & editing. AC: Data curation, Formal analysis, Investigation, Validation, Visualization, Writing – original draft. RM: Data curation, Formal analysis, Funding acquisition, Investigation, Project administration, Writing – original draft.
